# Post-Translational Modifications of Histones Are Versatile Regulators of Fungal Development and Secondary Metabolism

**DOI:** 10.3390/toxins14050317

**Published:** 2022-04-29

**Authors:** Aurelie Etier, Fabien Dumetz, Sylvain Chéreau, Nadia Ponts

**Affiliations:** INRAE, UR1264 Mycology and Food Safety (MycSA), F-33882 Villenave d’Ornon, France; aurelie.etier@inrae.fr (A.E.); fabien.dumetz@inrae.fr (F.D.); sylvain.chereau@inrae.fr (S.C.)

**Keywords:** chromatin, mycotoxins, metabolites, histone code

## Abstract

Chromatin structure is a major regulator of DNA-associated processes, such as transcription, DNA repair, and replication. Histone post-translational modifications, or PTMs, play a key role on chromatin dynamics. PTMs are involved in a wide range of biological processes in eukaryotes, including fungal species. Their deposition/removal and their underlying functions have been extensively investigated in yeasts but much less in other fungi. Nonetheless, the major role of histone PTMs in regulating primary and secondary metabolisms of filamentous fungi, including human and plant pathogens, has been pinpointed. In this review, an overview of major identified PTMs and their respective functions in fungi is provided, with a focus on filamentous fungi when knowledge is available. To date, most of these studies investigated histone acetylations and methylations, but the development of new methodologies and technologies increasingly allows the wider exploration of other PTMs, such as phosphorylation, ubiquitylation, sumoylation, and acylation. Considering the increasing number of known PTMs and the full range of their possible interactions, investigations of the subsequent Histone Code, i.e., the biological consequence of the combinatorial language of all histone PTMs, from a functional point of view, are exponentially complex. Better knowledge about histone PTMs would make it possible to efficiently fight plant or human contamination, avoid the production of toxic secondary metabolites, or optimize the industrial biosynthesis of certain beneficial compounds.

## 1. Introduction

Eukaryotic genomic DNA (gDNA) is organized in a dynamic structure, known as chromatin. Its fundamental unit is the nucleosome, i.e., ~146 base pairs of gDNA wrapped around a histone octamer containing two copies of each histone, H2A, H2B, H3, and H4 [1], also referred to as *core* histones. The interaction between the highly basic histones, due to numerous arginine and lysine side chains, and the negatively charged phosphate backbone of DNA maintains a charge neutralization that allows nucleosome stabilization [2]. A fifth histone, the *linker* histone H1, is not directly incorporated into the nucleosome, but interacts with it to ensure chromatin cohesion as well as contribute to chromatin dynamics [3]. The equilibrium of this structure leads to the formation of higher-order structures of chromatin, mainly represented by opened chromatin, euchromatin, amenable to transcription, and closed chromatin, heterochromatin, that is poorly or not transcribed. Classically, heterochromatin is qualified as *facultative* to refer to genomic regions that can dynamically adopt open or compact conformations, or as *constitutive* when genomic regions with a definitively compact conformation are considered. Literature regarding the biological functions of chromatin structure changes is diverse and abundant, and chromatin dynamics are largely described to be major players in a wide array of events, such as growth, differentiation, adaptation to changing environments, or genome stability and evolution.

Histone genes are tightly regulated; they are typically expressed during the replication phase of the cell cycle (see [4] for a review). Histones are lysine-rich, positively charged small globular proteins that are highly conserved across species [1]. Core histones are classically less than 150 amino acids in length, and 11–15 kDa in size [5]. They consist of a well-conserved ‘histone-fold’ domain, involved in histone–histone interactions to form the octameric structure around which the gDNA is wrapped, flanked by N- and C-terminal variable tails that stick out of the nucleosome particle, making them accessible to enzymes involved in the covalent deposition of numerous post-translational modifications (PTM) [2]. The global mass of the PTM decorating histone tails has been estimated to represent ~25% of the total histone mass [5]. Histone tails are fundamental for the secondary and tertiary structure of chromatin [6]. Among the variety of possible PTMs decorating histone tails, lysine acetylation and methylation, serine/threonine phosphorylation, and lysine ubiquitylation are the most commonly studied [2]. Thanks to rapid advances in equipment and methods, a more diverse catalog of PTMs is increasingly being considered [7,8], such as crotonylation, malonylation, succinylation, and propionylation. The various PTMs are deposited mostly on lysine (K), arginine (R), threonine (T), glycine (G), and alanine (A) residues of the N-terminal and C-terminal tails, but also within the histone-fold region [2]. Altogether, the combinations of PTM deposited form the ‘Histone Code’, which superimposes on the genetic code to regulate gene expression. Histone PTMs were shown to act synergistically or antagonistically, supporting the ‘Histone Code’ hypothesis and underlying its complexity [9]. The highly dynamic character of this code allows a rapid adaptation to environmental changes, thanks to the epigenetic modifier of the chromatin, i.e., writers, erasers, and readers: Writers add functional groups to histones, erasers remove them, and readers recognize PTMs and serve as anchors between PTMs and associated proteins, such as transcription factors, activators, repressors, or other proteins involved in the modulation of chromatin status [10]. The addition/removal of PTMs changes the energetic landscape of histones leading to their chemical structure alteration. Changes in the charges, size, or hydrogen bonding can alter histone–histone or histone–DNA interactions. The activity of writers, erasers, and readers dynamically modulates the chromatin state.

## 2. Chromatin Dynamics to Regulate Genomic Segments in Fungi

Studies focusing on yeasts have often pioneered investigations on chromatin structure dynamics. The budding yeast *Saccharomyces cerevisiae* and fission yeast *Schizosaccharomyces pombe* are well-studied and often qualified as model organisms because of the similarities between yeast and mammal protein encoding genes [11], with their unicellular eukaryotic status further facilitating extensive studies. The filamentous fungus *Neurospora crassa* is also considered as a model organism, with its multicellular status allowing a tighter link with higher eukaryotes. In addition, *N. crassa* shares numerous genes with potent phytopathogens while being non-pathogenic, making it a suitable model for a broad range of studies [12]. Fungal phytopathogens are particularly scrutinized because of the tremendous losses their presence in crops can cause, amounting to billions of dollars each year [13]. These losses can be the direct result of sharp falls in agricultural yields upon infection, or the indirect consequences of food/feed spoilage with mycotoxins, i.e., fungal secondary metabolites that are toxic for human and animals when ingested. For example, *Fusarium graminearum* is involved in small grain cereal field infections in most areas worldwide and produces type B trichothecenes such as deoxynivalenol (DON), causing nausea, vomiting, and immunosuppressive diseases; fumonisins mostly produced by *Fusarium verticillioides* and *Fusarium proliferatum* on corn may provoke human oesophageal cancer upon chronic exposure [14]. Despite important progress over the last couple of decades, particularly in the fields of genetics and genomics, current knowledge is not sufficient to elaborate mycotoxin management strategies that can guarantee the sanitary quality of cereal-derived food and feeds.

The production of secondary metabolites is largely dependent on environmental parameters, such as nutrient sources (carbon, azote), pH changes, or oxidative stress [15,16]. Non-essential for immediate survival, secondary metabolites can confer fitness benefits or play important roles in interacting with other organisms, such as bacteria, plants, insects, and other fungi (see review [16]). Additionally, these metabolites play major roles in adaptation to environmental changes [16]. Biosynthetic pathways leading to the production of secondary metabolites usually involve various enzymes, transcriptions factors, and transporters encoded by genes that are typically arranged in a biosynthetic gene cluster in fungal genomes, i.e., genes grouped at the same *locus* [16,17]. This clustered organization allows for a timely co-ordinated regulation of these neighbouring genes to control secondary metabolism activation or repression [18].

The genomic location of these clusters has been identified as a key factor of their regulation [15]. Indeed, they are, for the vast majority, located near to subtelomeric regions [15]. These regions are mainly constituted by facultative heterochromatin, implying a closed conformation which can be rapidly amenable to a relaxed one. This location is highly conserved between fungal species, highlighting the major role of chromatin conformation on secondary metabolism activation/repression. Among regulatory factors involved in the chromatin conformation modulation, PTMs represent major players enabling vast structural and biological effects, including chromatin status modulation, gene expression regulation, DNA damage control, and repair [19,20,21,22].

## 3. Post-Translational Modifications of Histones in Fungi

**Figure 1:**
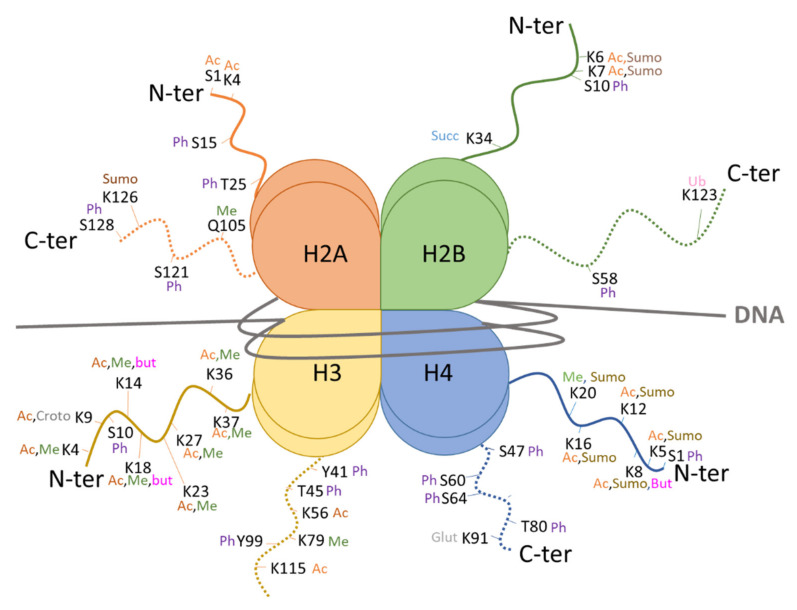
Histone post-translational modifications with a proposed function frequently reported in *S. cerevisiae*. Schematic representation of a nucleosome containing the DNA (in grey) wrapped around the canonical histones 2 × H2A, 2 × H2B, 2 × H3, and 2 × H4. The N- (N-ter) and C-terminal (C-ter) tails of these histones are decorated at specific residues (K, S, T, Q) with the following PTMs. Acetylation: Ac; methylation: Me; phosphorylation: Ph; succinylation: succ; sumoylation: Sumo; ubiquitylation: Ub; butyrylation: But; glutarylation: Glut. See Supplementary Table S1 for references.

The repertoire of histone PTMs in eukaryotes is vast and diverse, and the meaning of various histone PTM combinations may vary. Notably, the subject of histone acetylation and methylation in *S. cerevisiae*, and *S. pombe* to a lesser extent (reviewed in [23] for example), has been heavily treated. Figure 1 depicts a compilation of core histone PTM with experimentally proposed functions in *S. cerevisiae*. A variety of modifications decorate the N- and C-terminal tails, involved in diverse biological processes, such as acetylations, methylations, phosphorylations, sumoylations, ubiquitylations, butyrylations, and glutarylation. PTMs also decorate the histone-fold domain, which then affects the unwrapping dynamics and the stability of the nucleosome [2]. Modifications occurring within the histone-fold domain remain poorly documented.

However, these only represent the tip of the mountain, the repertoire of histone PTMs being constantly enriched by an expanding toolbox of proteomics approaches. Studying core histone PTMs is classically based on the targeted enrichment of a specific modification, mostly by techniques involving antibodies, which may result in non-specific binding on sites and cross-reaction [24]. Nowadays, mass spectrometry is considered the most appropriate method to identify PTMs, notably to identify novel ones (see review [24,25]). These approaches certainly contribute to rapidly enrich the repertoire of described PTMs. However, among these newly identified PTMs, very few of them only are linked to a function, and the vast majority of this functional studies concern the more familiar modifications, such as acetylation, methylation, and phosphorylation [26]. Studies have tried to provide keys for the understanding of such functions, notably by the development of thermodynamic models [26]. However, most functions associated to histone PTMs remain undemonstrated. Supplementary Table S1 illustrates the large diversity of post-translational modifications that have been detected (all methods included) in selected fungi: the yeasts *S. cerevisiae* and *S. pombe,* for which an abundance of reference data is available, and the human pathogen *Candida albicans*, the model filamentous fungus *N. crassa*, as well as the mycotoxin-producing phytopathogenic fungi *F. graminearum* and *A. nidulans,* both representative for phytopathogenic fungi.

**Figure 2:**
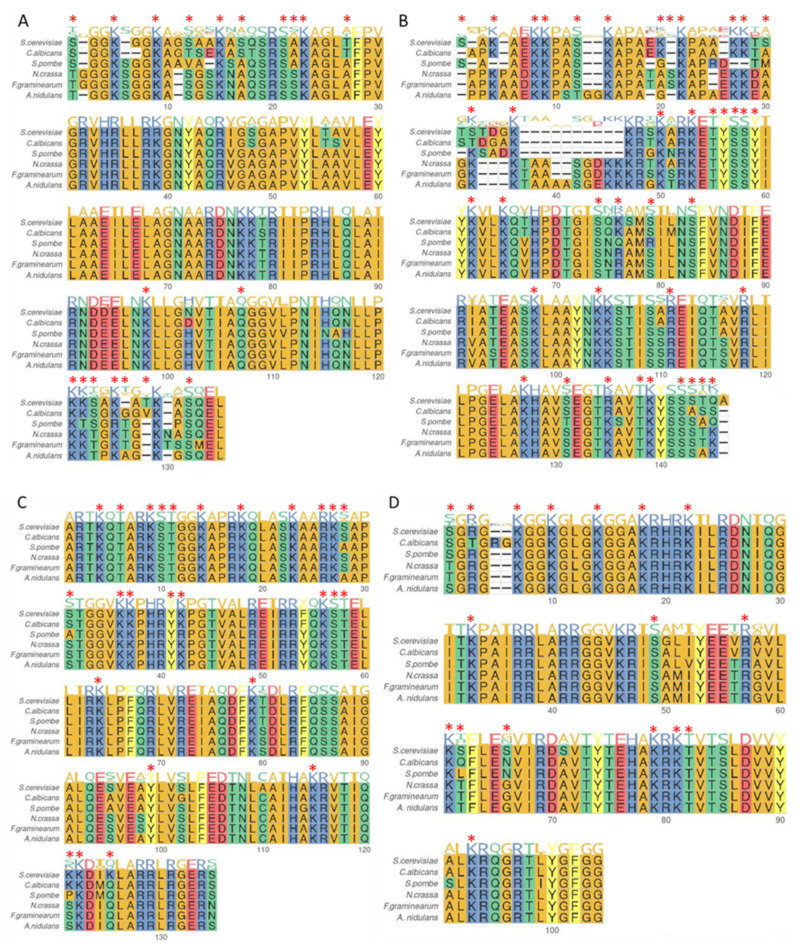
Protein sequence alignments canonical histones H2A, H2B, H3 and H4 in selected fungi. Initiator methionine was removed prior alignment with COBALT [28] and plotting with *ggmsa* 1.0.0 [29]. Background color reflect amino acid groups: positively charged = blue, negatively charged = red, polar uncharged = green, aromatic with hydrophobic side chain = yellow, others = orange. Sequence logo indicates sequence conservation for each position of the alignment. Red stars indicate positions with PTMs as in Supplementary Table S1. (**A**). H2A alignment of *S. cerevisiae* (YDR225W), *C. albicans* (C3_03910W_A), *S. pombe* (SPCC622.08c), *N. crassa* (NCU02437), *F. graminearum* (FGRAMPH1_01G26109), *A. nidulans* (AN3468) (**B**). H2B alignment of *S. cerevisiae* (YDR224C), *C. albicans* (C3_03900C_A), *S. pombe* (SPCC622.09), *N. crassa* (NCU02435), *F. graminearum* (FGRAMPH1_01G26111), *A. nidulans* (AN3469) (**C**). H3 alignment of *S. cerevisiae* (YBR010W), *C. albicans* (C1_04260W_A), *S. pombe* (SPBC1105.11c), *N. crassa* (NCU01635), *F. graminearum* (FGRAMPH1_01G14931), *A. nidulans* (AN0733) (**D**). H4 alignment of *S. cerevisiae* (YBR009C), *C. albicans* (C1_04240C_A), *S. pombe* (SPBC1105.12), *N. crassa* (NCU01634), *F. graminearum* (FGRAMPH1_01G14929), *A. nidulans* (AN0734).

Core histone proteins are often referred to as canonical histones because of their high level of sequence conservation, especially when related species are considered as illustrated Figure 2. Most of the time, amino acids that are divergent between species still belong to the same amino-acid family member. Additionally, positions for the preferential deposition of PTMs may also seem conserved (see Figure 2 and Supplementary Table S1), as well as often associated with a certain level of function conservation. For example, the methylation of the lysine 4 of histone 3 (H3K4me) and H3K36me are broadly described as acting as an active transcription marks, whereas H3K27me acts as a repressive mark (see [27] for a review). Various fungi are particularly suitable model organisms for studying chromatin dynamics and histone PTMs due to their highly conserved organisation of secondary metabolism genes in clusters typically located near subtelomeric regions, which are heterochromatin-enriched regions. The next sections detail and discuss the variety and the diverse functions of PTMs characterized in fungi to date, with a focus on toxin-producing phytopathogens.

### 3.1. Histone Acetylation, an Active Mark of Transcription

Histone acetylation may be the most studied PTM and is often associated with active gene expression [30]. Histone acetylation decreases the overall positive charge of histones, leading to weaker interactions between DNA and histones, and thus a more relaxed conformation of chromatin [30]. In addition, acetylation acts as docking sites for bromodomain-containing readers that can directly relax the chromatin, notably by labelling it to ATP-dependant chromatin remodellers [31]. This modification has been extensively characterized in eukaryotes as involved in the regulation of chromatin dynamics, transcription, cell cycle progression, apoptosis, differentiation, DNA replication, DNA repair, and nuclear import [32,33,34]. Histone acetylation is made through the transfer of an ethyl group from acetyl coenzyme A to histone lysines by histone acetyltransferases (HAT) [34]. HATs are grouped in five families, GNAT (GCN5-related N-acetyltransferases), MYST (MOZ, Ybf2/Sas3, Sas2, Tip60), p300/CBP, basal transcription factors, and nuclear receptor co-factors [35]. Conversely, histone deacetylases (HDAC) catalyze the removal of such acetylation. These HDACs are classified in three distinct classes, namely the “classical” HDACs, the silent information regulator 2 (SIR2) family, and the plant-specific HD2-type HDACs. Deacetylation increases the charge density of N-terminal tails of histones, which strengthen histone–DNA interactions, blocking access of the transcriptional machinery to DNA [36]. Classically, HDAC activity is tightly linked with transcriptional silencing. Interplays between HAT and HDAC lead to dynamic changes in chromatin structure and transcriptional activity. In filamentous fungi, the major role of the dynamic between histone acetylation/deacetylation on secondary metabolism is well documented, notably recently illustrated in *A. niger* by Li et al., 2019 [37]. Genome annotation coupled with phylogenetic analysis revealed seven possible HDACs. Deleting each HDAC individually led to modified secondary metabolism profiles, each one different from the other. This phenomenon will be thoroughly discussed below in other fungi.

#### 3.1.1. Molecular Events

Histone acetylation has been abundantly studied in yeasts with regard to transcriptional silencing, cell-cycle progression, and genome integrity. In *S. cerevisiae* the highly conserved HAT Gcn5, involved in the acetylation of K4, K9, K14, K18, K23, and K27 of histone 3 as well as K8 and K16 of histone 4 has been particularly studied [38]. In *S. cerevisiae*, the substitution of K8, K14, and K16 by a glutamine residue, thus inhibiting acetylation, had only minimal effects on cell growth [38]. However, the loss of Gcn5 in combination with these substitutions led to severe growth defects [39]. These results showed that Gcn5 is required for a full level of histone acetylation, not only restricted to these residues. Indeed, multiple acetyltransferases have been identified in yeast, notably Sas3 of the NuA3 complex responsible for H3K14ac and H3K23ac [40]. To study the combinatorial effect of these HATs, a double mutant for Gcn5 and Sas3 has been made and exhibited, then a non-viable phenotype. The combination of these results showed that the multiple HAT in yeast show redundancy of function. The construction of ΔGcn5 and ΔSas3 conditional mutants in temperature-shift assays, to bypass the lethality of the ΔGcn5ΔSas3 double mutant, exhibit, after a temperature shifting, a cell cycle progression arrest in G2-M. This arrest may be due to defective transcription of genes involved in the cell cycle progression or to defective changes in chromatin structure occurring normally during the G2-M transition [38,40]. The role of histone lysine acetylation in cell cycle progression is also mediated by H3K56ac, located in the histone H3 globular domain. Post-translational modifications in the histone globular domain classically influence DNA–histone interactions. Specifically, the deposition of H3K56ac by Rtt-109 and Asf1 in yeast leads to weaker interactions between the nucleosome and the DNA, allowing increased DNA accessibility at promoters during the activation of transcription [41]. Paradoxically, H3K56ac also promotes chromatin assembly in an Rtt106- and Caf1-dependant manner upon the yeast replicative phase. This opposing effect of H3K56ac can be explained by the energetically impossible way for Rtt106 to deposit (H3-H4)_2_ dimers onto replicating DNA directly.

#### 3.1.2. Developmental Consequences

Studies on histone acetylation have highlighted its major role in developmental events and in pathogenicity in fungi. In the yeast, *Cryptococcus neoformans*, an opportunistic human pathogen that causes meningoencephalitis in immunocompromised host [42], the deletion of Gcn5 leads to the loss of virulence in the murine model, linked to higher sensitivity of *C. neoformans* to elevated temperature (37 °C) and severe defects in capsule production [42]. In the same way, in the basidiomycete, *Ustilago maydis* responsible for corn smut disease, the loss of UmGcn5 (HAT) leads to a downregulation of genes known as involved in pathogenicity associated with a dramatic loss of virulence *in planta* [43]. In addition, this deletion leads to a long mycelial growth and the formation of a fuzz-like colony, whereas the wild type forms a yeast-like colony, exhibiting that UmGcn5 is important for *U. maydis* morphogenesis [44]. In the ascomycete *F. graminearum*, the loss of H3K14 and H3K4 acetylation in *ΔFgSas3* mutant, or H3K9, H3K14, H3K18, and H3K27 in *ΔFggcn5* mutant, showed a sharp growth rate reduction, respectively of 40% and 70% [45]. In addition, the loss of these acetylations leads to a dramatical reduction of conidiation in *ΔFgsas3* and to the complete loss of asexual reproduction ability in *ΔFggcn5*, suggesting specific functions for these acetyltransferases. In *Beauveria bassiana*, an entomopathogenic ascomycete, H3K56ac, deposited by the HAT Rtt109, is also required for normal growth and conidiation [46]. In a similar manner in *A. flavus*, Rtt109, responsible for both H3K9ac and H3K56ac, is also involved in growth, asexual reproduction, and in pathogenicity [47]. In addition to the role of histone acetylation in morphogenesis, asexual reproduction, and virulence, studies underlined their role in responses to environmental changes. For instance, in the ascomycete, *N. crassa*, H3K14ac deposited by Gcn5 is an active mark for light-induced gene expression [48]. Following light exposure, an increased abundance of H3K14ac mark in the *al-3* gene (light inducibility gene) has been observed concomitantly with *al-3* mRNA expression induction. In addition, the substitution of H3K14 by glutamine (which cannot be acetylated) leads to the loss of photo-inducibility, proving its function in light response [48]. In *F. graminearum*, FgSAS3 and FgGCN5 exhibited a role in the oxidative and osmotic stress sensitivity [45]. Altogether, these results highlight the important role of H3 lysine acetylation for normal development and ability to cope with environmental changes.

Conversely, proper histone deacetylation is also essential for development. For instance, in the phytopathogenic ascomycete *Magnaporte oryzae* that infects rice and wheat, H3K18ac deposited by Sas3 [49] is deacetylated by the recruitment of MoHos2 (HDAC part of Tig1 histone deacetylase complex) [50]. This deacetylation corresponds to a global change in gene expression levels, indicating that MoHos2 may be a master regulator [51]. The decrease of H3K18 deacetylation in the ΔMoHos2 mutant leads to a reduced vegetative growth, conidiation, and loss of pathogenicity [50]. Actually, MoHos2 plays a major role in regulating several developmental functions, such as conidiation, pathogenicity, histone modification, and cell cycle control, in different fungi, notably in *C. albicans*, in the ascomycete *Cochliobolus carbonus*, or in *U. maydis* [52,53,54]. As a whole, the highly dynamic deposition/removal of acetylations revealed a large spectrum of role in the development of yeasts, ascomycetes and basidiomycetes.

#### 3.1.3. Production of Secondary Metabolites

**Figure 3:**
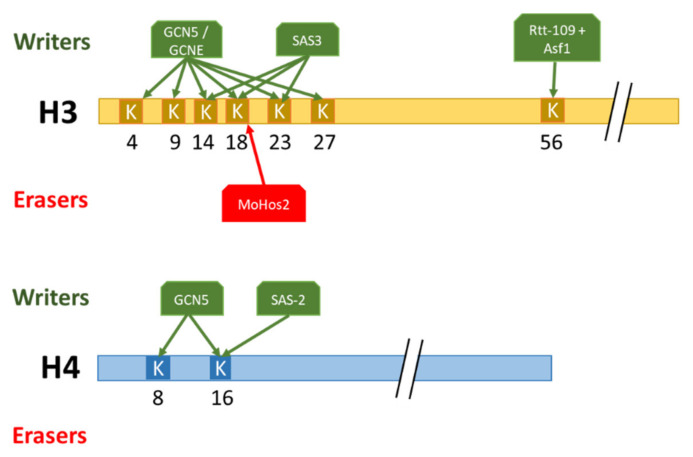
Summary of molecular actors involved in the deposition/removal of histone H3 and H4 acetylation. Lysine: K.

The involvement of chromatin structure changes in modulating the production of fungal secondary metabolites was first described in *A. nidulans* [55]. Since then, PTMs of histones have been characterized as having a major role in the regulation of secondary metabolism in other filamentous fungi [19]. In *A. nidulans*, the loss of GcnE (homologous to GCN5) leads to an impaired production of sterigmatocystin and terrequinone and to only 3% of penicillin production compared with the WT [56]. These results are consolidated with no or a highly reduced expression of the biosynthesis genes of these secondary metabolites. The activation role of acetylation on secondary metabolism production is highlighted by the demonstration in *A. nidulans*, that H3K9ac and H3K14ac deposited by GcnE have been localized by ChIP-seq in the promoter region of the sterigmatocystin biosynthesis positive regulatory gene *aflR* upon the induction of secondary metabolism production [57]. In addition, the loss of HdaA, a central contributor to total HDAC [58,59,60], exhibited an increase in sterigmatocystin (ST) and penicillin production correlated with a higher transcriptional level of genes contained in the two corresponding biosynthetic gene clusters in *A. nidulans*. In *F. graminearum,* the loss of acetylated H3K4, H3K9, H3K14, H3K18, and H3K27 by the deletion of FgGcn5 and FgSas3 leads to virtually no DON production concomitantly with a significant downregulation of genes contained in DON cluster [45]. In *A. parasiticus*, similar observations about the positive role of histone acetylation on secondary metabolite production have been made [61]. In this model, the enrichment of H4 acetylation during the induction of aflatoxin production permits the recruitment of *Aflr* to *Aflr*-binding sites located in promoter regions of the aflatoxin biosynthesis gene cluster, leading to a co-ordinated activation of aflatoxin production. By contrast, three *F. graminearum* PKS (polyketide synthases (PKS), involved in the biosynthesis of zearalenone (ZEA), fusarielin, and arsellinic acid, respectively, are up regulated in ΔFgSAS3 [62]. These contrasting results demonstrated the diversified role of acetylation, possibly depending on the combinatorial effect of histone lysine acetylation with neighbouring residues (Figure 3). These results underline the major role of histone acetylation/deacetylation in regulating secondary metabolism, notably by the rapid recruitment or removal of these marks to biosynthetic gene clusters. The particular dynamics between histone acetylation and methylation plays a major role in regulating secondary metabolite biosynthesis, e.g., H3K27ac/H3K27me3 responsible for activating/repressing DON production, respectively [45,63].

### 3.2. Methylation, a Highly Diverse Array of Functions

The role of histone methylation on chromatin structure remains unclear, despite much literature on the subject. From the biochemical point of view, histone methylation allows the tighter wrapping of histones tails around DNA, leading to a restricted access to DNA. In addition, this modification serves as docking sites for other proteins, especially those containing a chromodomain [64]. The major methylation sites are the basic amino acid chains of lysine and arginine residues. They can be associated with gene activity or gene repression depending on context, location, nature, and number of methylations: mono—(me), di—(me2) or tri-methylation (me3). Methylation involves the attachment of a methyl group to nitrogen atom in amino acid side chains and/or at the amino termini. Histone methyltransferases (HMTs) catalyse the transfer of a methyl group from a S-adenosylmethionine to the ε–amine on the side chain of lysine (KMT) or arginine residue (PRMT) [65]. Lysine can be mono-, di-, or trimethylated, whereas arginine can be only mono- or di-methylated [66]. There are two types of KMT: those containing the SET domain (Su(var)3-9, Enhancer-of-zeste and Trithorax) and those characterized by the absence of this domain. Within the SET-domain, there are four major proteins, namely KMT-COMPASS, SET2, spCLRC/NcDCDC, and PRC2, catalysing the di- and trimethylation of H3K4, H3K36, H3K9, and H3K27, respectively [23]. H3K4me and H3K36me are considered as active marks for gene expression [67], contrasting with H3K9me3 [68] and H3K27me3 [69] labelling gene expression repression. In addition, H3K9me3 has been further identified as a mark of constitutive heterochromatin, principally associated with centromeric heterochomatin, and H3K27me3 as a mark of facultative heterochromatin [69]. Catalyzing the removal of methyl groups decorating histones, the first histone demethylase (HDM) was discovered in 2004 [70]. HDM are grouped in two main families: lysine-specific demethylase (LSD) and Jumonji C (JmjC) [71]. Contrasting results about the role of methylation on several biological processes emphasize the complexity of this mark in fungi.

#### 3.2.1. Molecular Events

Methylation is involved in various molecular processes, such as DNA damage repair (DDR), genome integrity, chromatin status regulation, and recently the siRNA mechanism. In *F. fujikuroi*, mutants devoid of the KMT Ash1, responsible for the deposition of H3K36me3, were lacking two subtelomeric regions of chromosome I and X, suggesting a role of Ash1-dependant H3K36me3 on chromosome stability [72]. This genomic instability was not observed in double mutants *Δset2Δash1*, suggesting that subtelomeric deposition of H3K36me3 depends on Ash1 and requires the presence of Set2 [72]. Another example of the major role of histone PTMs on genome integrity is described in the yeast *S. pombe*. The deposition of H4K20me by SpSet9 is an essential mark associated with H2A phosphorylation for recruiting Crb2 (DNA damage checkpoint protein) to nuclear specific loci of DNA damage [73]. This example further illustrates combinatorial effects of two different histone PTMs in regulating a molecular process.

One of the most studied histone methylation marks may be H3K9me3, mostly known as involved in heterochromatin formation. This mechanism has been elucidated in *N. crassa*, in which NcDIM5 (H3K9 methyltransferase) has been identified as responsible for the deposition of H3K9me3 [74]. H3K9me3 is a binding site for Hp1 (Heterochromatin Protein 1) [75], allowing the recruitment of NcDIM-2 (cytosine methyltransferase) involved in DNA methylation. DNA methylation coupled to histone methylation are both essential for heterochromatin formation and its maintenance in *N. crassa* [76]. This mechanism has been studied in other filamentous fungi, and notably in *A. nidulans,* in which similar underlying mechanisms for heterochromatin formation are observed [57]. Contrasting with this model, a study in *F. graminearum* showed that the deletion of *FgHp1* leads to the loss of H3K9me3, demonstrating a modification of this sequential mechanism [77]. In *S. pombe,* the small interfering RNA (siRNA) pathway is involved in H3K9me3 positive feedback and in gene silencing inheritance [78]. Cells lacking either key siRNA or H3K9 methyltransferase Clr4 failed to maintain the inheritance of gene silencing. In addition, the decrease of H3K9 demethylation induced by the deletion of the demethylase *epe1* partially suppresses the requirement of siRNA for gene silencing. Here, the siRNA pathway and the deposition/removal of H3K9me3 are intertwined mechanisms for gene silencing inheritance.

#### 3.2.2. Developmental Events

In filamentous fungi, the role of histone methylation in developmental processes has been extensively studied. Four main histone methylations were mostly studied, H3K4me, H3K9me, H4K27me, and H3K36me for their major role in developmental and virulence regulation. H3K4me is typically described as an active gene mark. The reduction of H3K4me in *F. graminearum* leads to severe growth defects and decreased virulence [67]. In the ascomycete *Colletotrichum higginsianum*, the deletion of CclA required for the deposition of H3K4me3 leads to reduced mycelial growth, asexual sporulation, and spore germination, a decreased production of secondary metabolites production associated with a strongly reduced virulence on plants [79]. In *N. crassa,* H3K4me3 has been identified as playing a role in the normal clock gene expression, notably by regulating the proper expression of *frq* gene, which is a major gene associated with the regulation of circadian and light-system [80,81]. In addition, H3K4me3 is required for the acclimation of *N. crassa* under temperature fluctuation [82]. Moreover, the study of acclimation to fluctuating environments of several *N. crassa* strains with deficiency in histone methylation showed that H3K36me deposition/removal follows a rhythmic cycle, leading to the cyclic activation of the *frq* locus [82,83]. These results underline the role of H3K4me3 in setting up an adapted response to fluctuating environmental conditions. This common function suggests a crosstalk between H3K4me3 and H3K36me3 for a synergic control of gene expression. Similar to H3K4me3, H3K36me3 is generally associated with gene expression activation. For instance, the loss of H3K36me in *F. fujikuroi* strongly affects vegetative growth and conidiation and reduces its pathogenicity in planta [72]. In *N. crassa,* the loss of H3K36 mono-, di-, and tri-methylation leads to slower growth, conidial defect, and female sterility [84].

Regarding the heterochromatic mark H3K9me, roles seem to differ notably depending on the considered species. *N. crassa* ΔNcDim5 displays a moderately altered phenotype, showing slow and irregular growth [74]. Moreover, *F. verticilloides ΔFvdim5* mutant exhibited severe defects in conidiation and in perithecium formation [85]. In contrast, the reduction of H3K9me3 mark in *F. graminearum* did not exhibit phenotypic differences compared to the wild type [77]. Such diverging roles for histone PTMs according to the considered species can also be observed regarding H3K27me3. H3K27me is a hallmark for facultative heterochromatin [69]. In *F. graminearum* and *F. fujikuroi*, it has been shown that almost a third of their respective genome is enriched with H3K27me3 deposited by KMT6, suggesting its fundamental role in these fungi [63,86]. In *F. graminearum*, the lack of KMT6 leads to no more H3K27me3 and strong developmental defects, such as sterility, slow growth, an aberrant fate of germination, and reduced pathogenicity [63]. In *M. oryzae*, the same deletion leads to slower growth [87]. More dramatically, the loss of H3K27me3 in *F. fujikuroi* is lethal [86].

As mentioned earlier, histone arginine can also be modified by methylation, although this PTM is less documented than lysine methylation. In *A. nidulans* three protein arginine methyltransferases (PRMTs) have been identified: RmtA (arginine methyltransferase A) and RmtC involved in H4R3 methylations, and RmtB involved in the arginine methylation of H2A, H3, and H4 [88]. Δ*rmtA* and Δ*rmtC A. nidulans* mutants exhibited growth retardation under osmotic stress [89]. The same effects were observed for Δ*RmtC* mutants, although less pronounced. These observations are consistent with post-translational modifications of histones allowing a rapid and adapted response to environmental changes.

#### 3.2.3. Production of Secondary Metabolites

The role of histone methylation in the regulation of secondary metabolism in filamentous fungi has been scrutinized in various studies. In *F. graminearum* and *F. verticilloides*, H3K4me has been identified mainly as a transcriptional activator of biosynthetic gene clusters. For instance, the loss of *Set-1* in *F. verticillioides* and *F. graminearum,* involved in the deposition of H3K4me3, leads to a decrease of fumonisins and DON production, respectively, coupled with a downregulation of the expression of their respective biosynthetic gene clusters [67,90,91]. Contrasting with the steady state of active gene expression linked to H3K4me3, the lack of H3K4 methyltransferase CclA in the grass endophyte *Epichloë festucae*, in several *Aspergillus* species and in *C. higginsianum* leads to the induction of biosynthetic gene clusters [79,92,93,94]. In the same way, the loss of SET-1 in *F. fujikuroi* leads to an increased production of fusarins and bikaverin [90]. Altogether, these results could pinpoint a major species-dependent role of H3K4me in regulating the production secondary metabolites. These contrasting effects of H3K4me3 could be due to the low content of this modification in secondary metabolite clusters of certain species, notably in *F. graminearum* and *A. nidulans* [63,95]. In these species, the deletion of CclA could regulate the expression of secondary metabolite clusters in an indirect manner, explaining the dual role of H3K4me3 in both gene expression activation and repression [90].

H3K9me3 is associated with the repression of the biosynthetic gene cluster. In *A. nidulans*, ChIP-seq (Chromatin-ImmunoPrecipitation-sequencing) experiments demonstrated that H3K9me3 deposited by ClrD is associated with the silenced sterigmatocystin cluster [57]. In addition, deleting ClrD from *A. nidulans*’ genome leads to a global over-expression of secondary metabolite genes. In the same way, the loss of H3K9me3 in *F. graminearum* leads to higher levels of production of aurofusarin, involved in its red pigmentation [77]. In contrast, in the same study, Reyez-Dominguez and colleagues showed that the production of DON decreased in *F. graminearum* after the loss of H3K9me3. This decrease, contrasting with other observations in filamentous fungi, may be explained by a de-repression of a DON cluster repressor or a combinatorial effect with an unknown player.

Finally, H3K27me3 revealed itself as an important player in repressing the production of secondary metabolites [63]. In *F. graminearum* and *F. fujikuroi,* secondary metabolite genes expression was de-repressed in KMT6 lacking or knock-down mutants, including aurofusarin gene cluster, nine genes included in the fusarin C cluster, and some genes included in the DON cluster [63,86]. In the same way, the loss of ezhB in *E. fistucae*, involved in the deposition H3K27me3, leads to a de-repression of Lolitrem and ergot alkaloids biosynthesis clusters [96].

**Figure 4:**
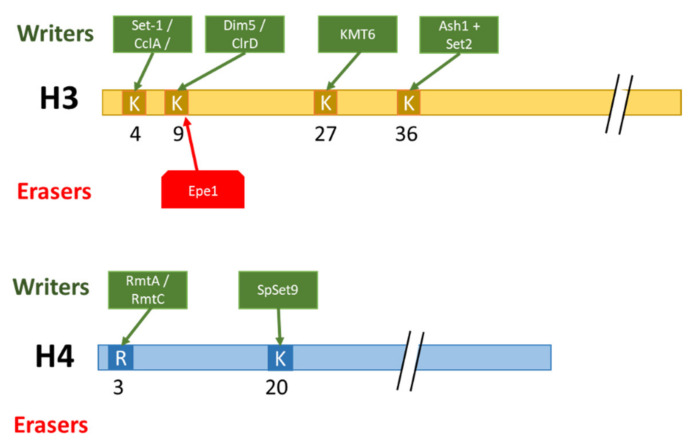
Summary of molecular actors involved in the deposition/removal of histone methylation. Lysine: K; Arginine: R.

As a whole, these examples (summarized in Figure 4) highlight the major role of histone methylation in regulating the secondary metabolism, notably by the rapid recruitment or removal of these marks to biosynthetic gene clusters. However, the functions of methylated marks seem to greatly vary depending on their nature, genomic context, including the consideration of other histone marks, and the considered species. The combinatorial aspects of histone marks are discussed in the ‘Histone Code’ section of this review.

### 3.3. Others PTMs

#### 3.3.1. Phosphorylation

Histone phosphorylation occurs on serine, threonine, and tyrosine residues, adding a negative charge, destabilizing the histone–DNA interaction. The dynamic character of deposition/removal of phosphorylation is catalysed by kinase proteins and phosphatase proteins, respectively [97]. All core histones can be phosphorylated and thereby regulate DNA accessibility [97], with an array of biological consequences described in eucaryotes in general and fungi in particular. Histone phosphorylation has been identified as involved in nucleosome unwrapping, notably through the H3Y41 and H3T45 phosphorylated residues [97]. This phenomenon leads to an opened conformation of chromatin. This relaxed structure facilitates the access to underlying DNA for factors involved in transcription, DNA repair, and replication. In *S. cerevisiae*, H3S10ph enhances the transcription, through its interaction with the Gcn5-dependant deposition of H3K14ac [98]. H3Y41ph has also been identified as involved in transcriptional regulation by downregulating the silencing of heterochromatin in sub-telomeric and centromeric regions in *S. pombe* [99]. H4S1ph was additionally shown to heavily decorate transcription start sites (TSS) of yeast genes particularly involved in late stages of sporulation [100]. Substituting of H4S1 by an alanine residue (non phosphorytable) caused a delay in the re-repression of genes involved in full-spore compaction that occurs for spore maturation, leading to impaired sporulation [101].

Phosphorylation of histones was particularly shown to play a role in regulating DNA repair mechanisms and cell cycle progression in yeasts. For example, in *S. cerevisiae*, H2AS122ph and H2AS129ph are essential for DNA damage response triggered after DNA double-stranded breakage, followed by cell cycle arrest [102,103]. Incidentally, in *B. bassiana,* the deletion of Hos2 responsible for H4K16ac, H3K56ac, and H2AS129ph is accompanied by spontaneous DNA damage, leading to genomic instability [46]. The mark H4S1ph, involved in the regulation of sporulation as previously cited, is also involved in DNA damage response during vegetative growth [104], decorating 1 kb-long regions spanning induced double strand breaks. Resuming the *S. cerevisiae* cell cycle further requires inactivating the DNA damage checkpoint, a step triggered by the deposition of H4T80ph [105]. During cell division, H2AS121ph was shown to be required for proper chromosome cohesion and stability in *S. pombe* [106] and *C. albicans* [107], respectively. In addition to its transcriptional and DNA repair functions, histone phosphorylation plays a major role in DNA replication [108]. For example, in budding yeast, H3T45ph is mainly deposited upon the S-phase of the cell cycle [109]. The loss of this residue is accompanied by an alteration of replication. Contrasting with this major role of phosphorylation in chromatin opening, some investigations also highlighted a role in chromatin compaction [108]. For instance, H4S1ph permits chromatin condensation [101]. Indeed, the loss of this phosphorylation in the mutant strain H4S1->A leads to less accessible chromatin, notably for ChIP experiments. In the same manner, H3 phosphorylation is related with the maximal chromosome condensation upon the pachytene during meiosis [110]. However, mechanisms underlying this role in DNA compaction remain unclear. This phenomenon could be explained by the fact that H4 tails bridge between one nucleosome to the acidic patch of the other [111]. The compaction function of these histone phosphorylations may be due to inter-nucleosomal interaction more than DNA–histone interaction. In *F. graminearum*, mass spectrometry-based phosphoproteome analyses showed that H4S47 is a target for phosphorylation [112], a modification involved in transcriptional reprogramming upon osmotic and heat stress in yeast [113].

#### 3.3.2. Ubiquitylation

Histone ubiquitylation consists of covalently attaching a small protein called ubiquitin (8.6 kDa) to lysine chains [114]. This adding requires three separate enzymatic activities. The first step leads to the formation of a thioester bond between the C-terminal end of ubiquitin and the catalytic cysteine of an ATP-dependant ubiquitin-activating enzyme E1. Then, the ubiquitin conjugates with a cysteine residue of a ubiquitin-conjugating enzyme E2 before being transferred to a lysine residue of histones by a ubiquitin-protein isopeptide ligase E3 that ensures substrate specificity [114]. Ubiquitylation is reversible. The removal of ubiquitin is catalyzed by deubiquitylases (DUBs), which are important cysteine proteases or metalloproteases that antagonize ubiquitylation and recycle ubiquitin moieties [114]. Histone residues can only be mono-ubiquitylated, which targets them to proteasome-independent signalling pathways. Ubiquitylation is typically involved in gene transcription regulation, nucleosome assembly and stability, and in the regulation of developmental events.

One of the most studied histone ubiquitylations in fungal species is H2BK123ub, highly conserved from yeast to human (H2BK120ub) [115,116]. The dynamic of deposition and removal of H2BK123ub is ensured respectively by Bre1 [117,118] and Ubp8 (subunit of the SAGA complex) [119]. A large number of studies about the role of H2BK123ub have pinpointed its major function in diverse biological functions, such as transcription regulation, telomere silencing and maintenance, nucleosome assembly and stability, DNA replication, chromosome segregation, and in developmental events. In *S. cerevisiae*, H2BK123ub is tightly linked to gene transcription activation [115]. This transcriptional activation is associated with the dynamic character of H2BK123 ubiquitylation. Indeed, the activation of transcription is due to the transient location of the Bre1 to RNApolII promoter of constitutively transcribed genes [119,120,121], permitting the recruitment of Rad6 (E2) associated to a transcriptional co-activator function [122]. Supporting this idea, the substitution of H2BK123 in H2BR123—non ubiquitytable—strains has been performed and studying the expression level of two genes, well-defined as regulated by chromatin status, GAL1 and SUC2, revealed a fourfold reduction of *GAL1* mRNA levels and 40% of *SUC2* mRNA levels [119]. On the contrary, the deubiquitylation of H2Bub leads to a more condensed chromatin structure [116] and reduced gene expression levels [123]. By contrast, H2BK123ub has also been associated with telomere silencing and maintenance [124,125]. These contrasting results show the opposite role of the same histone ubiquitylated mark, notably depending on the combinatorial effect with other adjacent PTMs, such as histone methylation [124]. *S. cerevisiae* H2BK123ub is further involved in modulating nucleosome occupancy through the chromatin remodeller Chd1, involved in the maintenance of a high level of H2B monoubiquitylation, notably towards the 3′ ends of genes [126]. H2BK123ub increases histone occupancy by reducing H3 solubility, leading to increased levels of H3 incorporation [127]. This nucleosome stabilization by H2BK123ub contributes notably to the progression of the replication fork [128]. In addition, H2Bub has been identified as playing a role in developmental events in yeast. In *S. cerevisiae*, the substitution of H2BK123ub and adjacent lysine residues, avoiding the ubiquitylation to others neighbouring sites, showed mitotic and meiotic defects [129]. In *C. neoformans*, decreased levels of H2BK123ub in *Δbre1* mutants led to growth defects and reduced self-filamentation correlated with the upregulation of 27 genes involved in filamentation suppression [130]. A function of H2BK123ub in *A. alternata,* a plant necrotrophic fungus, was revealed by the deletion of AaBre1 that caused growth retardation, decreased conidiation, and reduced virulence when infecting *Chrysanthemum morifolium* [131]. As a whole, these results underline the major role of H2Bub in developmental processes.

#### 3.3.3. Sumoylation

SUMO (small ubiquitin-related modifier) is a 10 kDa ubiquitin-like modifier that, similar to ubiquitin, can covalently bind lysine side chains of numerous proteins, including histones. In *S. cerevisiae* (and computational analyses indicate it is also the case in other fungi [132]), there is only one known SUMO protein, namely SMT3. Similar to the cascade of events leading to the attachment of ubiquitin moieties to a target lysine side chain, the deposition of SUMO requires the sequential intervention of E1, E2, and E3 enzymes. However, unlike ubiquitylating components that consist of several E1, E2, and a wider variety of E3 enzymes, SUMOylation machinery was found to consist of a single E1 (*Aso1*-*Uba2* in yeast) and E2 (*Ubc9* in yeast) that combine to a handful of known SUMO E3 enzymes (*Siz1*, *Siz2*, *Mms21,* and *Zip3* in *S. cerevisiae*) and a few other candidates with, to date, no demonstrated activity (see [132] for a review). Two SUMO proteases, catalyzing the removal of the SUMO moiety, complete the set, referred to as Ulp1 and Ulp2 in *S. cerevisiae* [133]. SUMOylation can occur on a variety of proteins including histones (see [134] for a review). In. the yeast *S. cerevisiae*, all the four canonical histones can be sumoylated [135]. H2A is sumoylated at position 126, H2B is sumoylated at K6/K7 or K16/K17, and H4 is sumoylated at K5, 8, 12, 16, and 20 [135]. Histone sumoylations are involved in both transcriptional activation and repression [135,136]. Histone sumoylations destabilizes nucleosomes, thus contributing to an opened state of chromatin supported by the enhanced affinity of the RSC (remodelling the structure of chromatin) complex, involved in a relaxed conformation of chromatin, for H2Bsumo-containing nucleosome [137]. Contrasting with this clear destabilizing effect of histone sumoylation on the chromatin state, its role in transcriptional activation/repression is not entirely elucidated. On one hand, H2B sumoylation is linked to actively transcribed regions [136]. On the other hand, studies have highlighted the role of histone sumoylation on gene expression repression [135]. For instance, the presence of sumoylated H2B is correlated with low levels of expression of *GAL1* in *S. cerevisiae*. Furthermore, it has been shown that sumoylated H2B are enriched near telomeric repeats, i.e., un-transcribed regions [135,138]. These contrasting observations have been partially explained by the combination of sumoylation with others PTMs, such as histone methylation, acetylation, and ubiquitylation. Evidence suggests that sumoylation acts as a fine-tuning controller of gene expression. For instance, the tight link between H4 sumoylation and the active mark of gene expression H3K4me3 has been demonstrated [139]. The deposition of H4sumo enhances the activity of the H3K4 demethylase LSD1, thus promoting gene expression silencing. Histone sumoylation further promotes the activity of Set-3 histone deacetylase complex involved in the deacetylation of 5′ end of genes to prevent the initiation of spurious transcripts [140]. In addition, histone sumoylation and histone acetylation seem to localize in a mutually exclusive manner upon gene activation [135]. The same opposite profile between histone sumoylation and ubiquitylation at telomeric regions has been observed [135]. The biological signification of histone sumoylation in filamentous fungi remains to be elucidated.

#### 3.3.4. Lysine Acylation

Recent proteomics approaches allowed the identification of a large group of non-acetyl histone acylation PTMs (see [141] for a review). This group includes propionylation, butyrylation, crotonylation, 2-hydroxyisobutyrylation, succinylation, and malonylation. The deposition of these modifications requires acyl-CoA groups produced from several metabolic pathways [142]. Most of these marks have been initially identified by mass spectrometry analyses in budding and fission yeast. In other fungi, these PTMs have not been extensively studied yet. Although non-acetyl histone acylations are usually catalyzed by “classical” acetylation modifiers, the mechanisms underlying the dynamics of their deposition and removal on histone remain poorly understood. One of the rare examples is the identification of Gcn5 and Esa1 involved in the crotonylation of H3K9, K14, and K23 in *S. cerevisiae*, with contrasted functions in both activating and repressing gene transcription [143].

Succinylation and lysine malonylation can also decorate histone lysine [144]. These two modifications induce virtually the same charge shift as phosphorylation in histone residues, and their analysis requires highly accurate mass spectrometry systems [145]. Seven sites of lysine succinylation have been identified in *S. cerevisiae*: H2AK13, H2AK21, H2BK34, H2BK46, H3K79, H4K31, and H4K77 [144]. The location of these PTMs in the core domains of the histones, which closely interact with DNA, may suggest a role of succinylation in modulating histone–DNA interaction. Their functions have been studied by the substitution of modified lysine by arginine (R) or alanine (A) to prevent succinylation, or by glutamic acid (E) or aspartic acid (D) to mimic constitutive lysine succinylation [144]. Most of these substitutions had no effect on *S. cerevisiae* phenotype. Only the H4K77E mutant displayed a loss of gene silencing at telomeres and rDNA, supporting a function of H4K77succ on stabilizing nucleosome assembly [146]. Two sites of lysine malonylation have been identified in *S. cerevisiae*, H2AK119 and H3K56 [144]. Malonylated H3K56 seems to play a role in cell viability [144]. Recently, the *novel* PTM lysine 2-hydroxyisobutyrylation has been detected on H4K8 in *S. cerevisiae* [147]. A peculiarity of this mark is that its kinetics of deposition/removal seems to entail metabolic feedback control loops involving glucose homeostasis genes [147]. In addition, it has been shown that H4K8A mutants, eliminating 2-hydroxysobutyrylation, are more sensitive to oxidative stress than the wild type.

The rapidly growing body of data relative to lysine acylation, enabled notably by high accuracy mass spectrometry analyses, underlines their frequent occurrence and the important character of these new PTMs. Notably, sirtuin HDAC enzymes, which are major regulators of primary and secondary metabolism in fungal species [148,149,150,151,152], also regulate the removal of non-acetyl lysine acylation, such as malonylation, succinylation, butyrylation, β-hydroxybutyrylation, propionylation, and crotonylation. [153]. This relationship between sirtuins and lysine non-acetyl acylation could pinpoint a major impact of these modifications on fungal metabolism.

## 4. The Histone Code

The ‘Histone Code’ hypothesis was introduced as a counterpart to the ‘genetic code’ of the DNA to describe the nucleosomal reshuffle with post translational modifications twenty years ago by Strahl and Allis [9]. They support the original idea that the presence of specific histone marks, and in some cases, possible combinations of histone marks, provides information to regulatory proteins that interpret isolated or combined marks to influence physiological or pathological functions. The Histone Code has been put forward to explain these combinations which have the potential advantage of providing increased robustness through cooperation and redundancy of combining the histone marks for the organisms [154]. This code would provide a necessary degree of proofreading so that the level of gene transcription could not be inappropriately modulated insofar as the loss of one mark would only have a minor effect on the associated phenotypes. Further, while most well-documented histone modifications and their impacts on chromatin are conserved and play a similar functional role across eukaryotes, it is increasingly clear that the Histone Code is not totally universal, and this underscores the need to avoid general conclusions obtained from only one organism [155].

The deciphering of the code, and the crosstalk amongst the underlying PTMs, is further complicated for the following main reasons. First, several studies have shown that the same residue can undergo different modifications, e.g., the H3K9 target can classically be either methylated or acetylated. The transcriptional consequences of these marks are sometimes opposed as acetylation of this residue is associated with transcriptional activation and methylation often, but not always, with repression of genes. Secondly, the Histone Code is versatile as combinations of PTMs can be altered by several internal or external stimuli. These lead to rapid changes in histone PTM patterns and to rapid adaptation to environmental modifications, particularly through their role in the coordination of fungal development and SM production [16,156]. Third, the exchange of canonical histones with histones variants, which differ slightly in their amino acid sequence and which are decorated with PTMs, increase the complexity of the structure and function of chromatin. Lastly, technical difficulties to elucidate the growing number of PTMs interactions, present numbers of challenges in particular because of their hydrophilic properties, their low abundance, and their lability. The fact that their presence may affect the cleavage of protease and that the histone composition is enriched in basic amino acids, which prevents some trypsin digested peptide from being analyzed by mass spectrometry, also complicates their identification.

Among the approaches to identify PTMs, the bottom-up mass spectrometry approach, using trypsin digestion, is still the most widespread used today to identify co-occurring PTMs in very short peptides ranging from four to 20 amino acids [157]. Although this method has the advantage of being significantly more sensitive than the other existing approaches and successfully used in identifying some novel modifications on histones [158], it seems not particularly well suited for the characterization of simultaneously occurring distant PTMs that would be present in different tryptic peptides. The middle-down approach, in which proteins are digested into peptides commonly in the 20–100 amino acids range, is emerging as an attractive alternative. In this special application of bottom-up proteomics, different digestion combinations are employed to obtain longer peptides than those formed by trypsin digestion [159]. This approach has the advantage of increasing the probability to detect multiple co-occurring PTMs in one peptide thereby allowing measurement of medium-range intra-protein PTM crosstalk. However, this technique, which requires an in-silico analysis before the experimentation to determine the best possible digestion enzymes combination, remains inefficient to study the long-range histone PTMs co-existence, which represents a technical challenge. To bypass this issue, the top-down approach consists in studying the entire protein, which is regarded as the most suitable method to investigate the combinatorial language of distant histone PTMs. This field was largely pioneered using histones, because these proteins are particularly amenable to top-down analysis due to their small size (11–23 kDa) and their relative high abundance [160]. The top-down analysis provides information about the stoichiometry of PTMs and makes it easier to differentiate histones variants and isoforms. However, the top-down approach is not always successful for the comprehensive characterization of the Histone Code because it remains relatively insensitive and require large amounts of samples [161]. Moreover, this application cannot be used routinely, because it requires adapted extensive prefractionation to enable the comprehensive analysis of as many proteoforms as currently possible [162]. Since top-down analysis cannot be currently considered as a true sensitive high-throughput proteomic method, it is recommended to combine it with compatible bottom-up strategies to obtain deep proteome coverage. Nonetheless, these methods for PTMs discovery must be associated with other functional approaches, such as ChIP-seq and RNA-seq, for the elucidation of their role [163].

Due to this complexity, very rare examples of histone PTM combinations, and even fewer examples concerning their biological functions, are available. In the literature, two different types of PTMs crosstalk have been proposed, mostly in yeasts: the “cis-effect” implicated in short-range interactions, and “the trans-effect” involved in long-range interactions. Both are mediated through the coordinated actions of PTM Writers, Erasers, and Readers. The cis-effect is described as a “communication” of adjacent modifications within the same histone tails [164]. For example, in the fission yeast, it has been demonstrated that Clr4 is associated with the ubiquitin E3 ligase Cul4 to form the CLR4 methyltransferase complex (CLRC) [165]. This complex preferentially ubiquitylates H3K14, and then promotes the methylation of H3K9. Trans-effects design cross-communications between different histone marks located within the same nucleosome [166]. For example, H2BK123ub is essential for the deposition of H3K4me2/me3 and H3K79me2/me3, which in turn affect gene expression [167,168].

To date, studies that explore the true combinatorial nature of the Histone Code remain scarce, which may seem paradoxical considering the growing number of identified histones PTMs with the advent of highly accurate mass spectrometry. Indeed, the complexity of these methods and the necessary expensive high-end instrumentation which somewhat limits their widespread use, render this kind of approach still under-exploited [169], especially since no single approach alone is able to support a complete combinatorial mapping of histone PTMs. Studying the diversity of histone PTM combinations and elucidating their relationships vs. their biological functions remains a challenge.

## 5. Conclusions

Fungi are major producers of secondary metabolites, which are incredibly structurally diverse molecules. Many of them are of considerable interest for pharmaceutical drug development. In contrast, some of these secondary metabolites represent a huge health hazard. In fact, just few fungal human pathogens are responsible for almost 1.5 million deaths per year [170]. Antifungal therapy mainly relies on fungistatic or fungicidal strategies. There are only four different chemical families of active compounds: polyenes, azoles, echinocandins, and flucytosine. In the medical field, the use of these compounds remains limited because of their toxicity in patients, the emergence of drug-resistant species and adverse drug–drug interactions [171]. In an agricultural consideration, there is an enhancement of fungal and mycotoxins contamination due to climate changes [172]. All accumulated knowledge about the major role of histone PTMs in regulating primary and secondary metabolism in fungi points towards promising developments of new antifungal strategies targeting these histone PTMs. Numerous chemicals are already known to activate or repress writers or/and erasers of histone acetylation (see review [173]). Among HAT inhibitors used for antifungal strategies, we can cite the Cyclopentylidene-[4-(4-chlorophenyl)thiazol-2-yl) hydrazone (CPTH2) that has fungicidal activity against *Candida* species [174]. This compound targets the GCN5 HAT network ([175] and the present review) with broad range of effects and targets. HDAC inhibitors are also used in antifungal therapies [173]. For instance, sodium butyrate and the trichostatin A (TSA) showed antifungal effects in *C. neoformans* by reducing its virulence, modifying its morphogenesis and leading to a premature cell-cycle arrest [59]. TSA also possesses an antifungal effect on *C. albicans*, *Aspergillus* species, and *M. oryzae* [59,176,177,178]. Nonetheless, the emergence of resistant strains against antifungal agents is a major concern. Histone acetylation plays a role in drug resistance in fungal species, marking the activation of resistance genes (see review [173]). One way to bypass resistance to certain antifungal agents is to use them synergically with other treatments. For example, Hos2 inhibitor MGCD290 can be used in combination with posaconazole to decrease the resistance in resistant strains of *F.* spp., *Zygomecetes* spp. and *C. glabrata* [179]. It also permits to sensitize filamentous fungi (such as *Aspergillus* spp., *Fusarium* spp., and *Zygomycete* spp.) to fluconazole, which showed no antifungal capacity on these species when tested alone [179]. To date, only HAT/HDAC inhibitors are histone PTM modifiers that are used as antifungal agents. Nevertheless, the potential function of the SET1-dependant H3K4me in the drug resistance of *C. glabrata* to azoles and of *S. cerevisiae* to Brefeldin A has been highlighted [180,181]. The wide variety of histone PTMs and their potential to be targeted in antifungal treatments represent a promising field that remains largely un-investigated.

The plurality and diversity of histone PTMs and their cross-interaction identification and understanding in filamentous fungi represent a challenging issue to be addressed. Deepening studies about “classical” PTMs, such as acetylation and methylation, coupled with studies about “non-classical” PTMs, such as lysine acylation, would allow a better understanding of these PTMs, their interactions, and their role in filamentous fungi. Recent views of the dynamics of histone PTMs deposition and removal underline a possible role of chromatin to store chemical groups, such as acetate derived from acetyl-CoA, to be released when the cell needs it (discussed in [182]). For example, histone deacetylation may serve to release acetate upon starvation, to renew the pool of acetyl-CoA for metabolism (reviewed by [183]). Several pieces of evidence are compatible with this hypothesis, including a tight link between Acyl-CoA content and chromatin regulation (reviewed in [184]). This balancing situation can be viewed as a competition of metabolism with histone acylation [185,186,187]. As a whole, understanding the Histone Code related to secondary metabolism regulations may allow the development of optimized approaches to fight against fungal contaminations or to manage the development of new pharmaceutical drugs.

## Data Availability

The data presented in this study are available in this article and Supplementary Materials.

## Supplementary Materials

The following supporting information can be downloaded at: https://www.mdpi.com/article/10.3390/toxins14050317/s1, references [19,21,39,45,56,57,63,67,80,88,89,95,100,101,102,103,105,106,107,110,112,117,118,119,123,129,133,135,136,138,144,155,188,189,190,191,192,193,194,195,196,197,198,199,200,201,202,203,204,205,206,207,208,209,210,211,212,213,214,215,216,217,218,219,220,221,222,223,224,225,226,227,228,229,230,231,232,233,234,235,236,237,238,239,240,241,242,243,244,245,246,247,248,249,250,251,252,253,254,255,256,257,258,259,260,261,262,263,264,265,266,267,268,269,270,271,272,273,274,275,276,277,278,279,280], Table S1: Histones and their post-translational modification associated with *S. cerevisiae, S. pombe, C. albicans N. crassa, F. graminearum and A. nidulans*.
