# Supplementary material for: Post-Translational Modifications of Histones Are Versatile Regulators of Fungal Development and Secondary Metabolism
Source: Toxins (Basel). 2022 Apr 29;14(5):317. doi: 10.3390/toxins14050317 (PMC9145779; doi:10.3390/toxins14050317)
Supplement: Supplementary file 1 [file toxins-14-00317-s001.zip › toxins-1681294-supplementary.pdf]

Supplementary Materials: Post-Translational Modifications of Histones Are Versatile Regulators of Fungal Development and Secondary Metabolism

Aurelie Etier, Fabien Dumetz, Sylvain Chéreau and Nadia Ponts

Table S1. Histones and their post-translational modification associated with S. cerevisiae, S. pombe, C. albicans N. crassa, F. graminearum and A. nidulans.

HISTONE	*SPECIES*	RESIDUE[1]	PTM	REFERENCES
H2A	*S. cerevisiae*	S1	Acetylation<br>Phosphorylation	[188,189]<br>[190]
	S. cerevisiae<br>*S. pombe*	K4	Acetylation	[191]<br>[192]
	F. graminearum	K5	Acetylation	[193]
	S. cerevisiae	K7	Acetylation	[191,194]
	S. pombe	K8	Acetylation	[192]
	N. crassa	K9	Acetylation	[195]
	S. cerevisiae	S10	Phosphorylation	[190]
	S. cerevisiae<br>*S. cerevisiae*	K13	Acetylation<br>Succinylation	[196]<br>[144]
	S. cerevisiae	S15	Phopshorylation	[197]
	C. albicans	S18	Phosphorylation	[198]
	S. cerevisiae<br>*S. pombe*	S19	Phosphorylation	[199]<br>[200]
	S. cerevisiae	K21	Succinylation<br>Ubiquitylation	[144]<br>[201]
	N. crassa	S21	Phosphorylation	[202]
	S. cerevisiae	T25	Phosphorylation	[203]
	S. cerevisiae	K96	Acetylation<br>Ubiquitylation	[204]<br>[201]
	S. cerevisiae<br>*S. pombe*<br>*C. albicans*	Q105	Methylation	[205]<br>[205]<br>[205]
	S. cerevisiae<br><br>*S. pombe*	K119	Acetylation<br>Malonylation<br>Ubiquitylation	[204]<br>[144,206]<br>[135]
	S. pombe	T120	Phosphorylation	[200]
	S. cerevisiae<br>*S. pombe*<br>*C. albicans*	S121	Phosphorylation	[199,102,106]<br>[207]<br>[107]
	S. cerevisiae<br>*S. pombe*	K123	Acetylation	[208]<br>[192]
	S. pombe	T124	Phosphorylation	[200]
	S. cerevisiae<br>*N. crassa*	T125	Phosphorylation	[209]<br>[210]
	S. cerevisiae	K126	Acetylation<br>Sumoylation	[208]<br>[135]
	S. cerevisiae<br>*S. pombe*<br>*C. albicans*	S128	Phosphorylation	[199,211,103]<br>[212]<br>[198]

	N. crassa	S130	Phosphorylation	[210]
	S. cerevisiae<br>*S. pombe*	S1	Phosphorylation	[190]<br>[200]
	N. crassa	K3	Acetylation<br>Methylation	[195]<br>[195]
	S. pombe	K5	Acetylation	[192]
	S. cerevisiae	K6	Acetylation<br>Sumoylation	[135]<br>[133,135,136,138]
	S. cerevisiae<br>*N. crassa*	K7	Acetylation<br>Sumoylation<br>Acetylation<br>Methylation	[135]<br>[13521,133,136]<br>[213]<br>[213]
	N. crassa	K8	Acetylation	[195]
	S. cerevisiae	S10	Phosphorylation	[214–216]
	S. pombe	K10	Acetylation	[192]
	S. cerevisiae	K11	Acetylation	[135,194,217,218]
	N. crassa	S11	Phosphorylation	[202]
	N. crassa	K12	Acetylation	[195]
	S. pombe	K15	Acetylation	[192]
	S. cerevisiae	K16	Acetylation<br>Sumoylation	[135,194,217,218]<br>[135]
	S. cerevisiae	K17	Acetylation<br>Sumoylation	[219]<br>[133,135,136]
	N. crassa	S18	Phosphorylation	[202]
	N. crassa	K19	Acetylation	[195]
	S. cerevisiae	K21	Acetylation<br>Butyrylation	[219]<br>[219]
**H2B**	*S. cerevisiae*	K22	Acetylation<br>Methylation	[219]<br>[219]
	S. cerevisiae	S24	Phosphorylation	[190,194]
	S. cerevisiae	S26	Phosphorylation	[220]
	N. crassa	K28	Acetylation	[195]
	N. crassa	K29	Acetylation	[195]
	S. cerevisiae	K30	Acetylation	[196]
	S. cerevisiae	K34	Succinylation	[144,221]
	S. cerevisiae	K37	Methylation<br>Succinylation	[219]<br>[144]
	S. cervisiae<br>*C. albicans*	T39	Phosphorylation	[222]<br>[198]
	S. cerevisiae	Y40	Phosphorylation	[222]
	S. cerevisiae<br>*C. albicans*	S41	Phosphorylation	[223,224]<br>[198]
	S. cerevisiae<br>*C. albicans*	S42	Phosphorylation	[199]<br>[198]
	N. crassa	K44	Methylation	[213]
	S. cerevisiae	K46	Ubiquitylation	[201]
	F. graminearum	T47	Phosphorylation	[112]
	N. crassa	Y47	Phosphorylation	[202]
	N. crassa	S48	Phosphorylation	[202]
	S. cerevisiae	K49	Ubiquitylation	[201]
	N. crassa	Y50	Phosphorylation	[202]
	N. crassa	K53	Acetylation<br>Methylation	[213]<br>[213]
	N. crassa	K56	Acetylation	[213]

			Methylation	[213]
	S. cerevisiae	S58	Phosphorylation	[225]
	S. cerevisiae	K60	Ubiquitylation	[201]
	C. albicans	S63	Phosphorylation	[198]
	A. nidulans	S77	Phosphorylation	[226]
	S. cerevisiae	K82	Ubiquitylation	[201]
	S. cerevisiae	K88	Ubiquitylation	[201]
	N. crassa	K89	Methylation	[213]
	N. crassa	K95	Acetylation	[213]
			Methylation	[213]
	N. crassa	R102	Methylation	[213]
	N. crassa	R109	Methylation	[213]
	S. cerevisiae	K111	Ubiquitylation	[201]
	S. cerevisiae	S115	Phosphorylation	[199]
	N. crassa	K118	Acetylation	[213]
	S. pombe	K119	Ubiquitylation	[227]
	S. cerevisiae	T122	Phosphorylation	[201]
	N. crassa	S122	Phosphorylation	[202]
	S. cerevisiae	K123	Ubiquitylation	[117-119,123,129,201]
	S. cerevisiae	S126	Phosphorylation	[201]
	N. crassa	K126	Methylation	[213]
	S. cerevisiae	S127	Phosphorylation	[201]
	S. cerevisiae	T128	Phosphorylation	[228]
	N. crassa	K136	Methylation	[213]
	S. cerevisiae	K4	Acetylation	[191]
			Methylation	[155,229-234]
	S. pombe		Methylation	[235,236]
	N. crassa		Acetylation	[195]
			Methylation	[80]
	F. graminearum		Methylation	[67]
			Acetylation	[193]
	A. nidulans		Methylation	[19,95]
	S. cerevisiae	T6	phosphorylation	[190]
	S. cerevisiae	K9	Acetylation	[39,155,194,230,237]
			Crotonylation	[238]
	S. pombe		Methylation	[240]
			Acetylation	[241]
**H3**	*C. albicans*		Acetylation	[195]
	N. crassa		Methylation	[242]
	F. graminearum		Methylation	[45]
			Acetylation	[45]
	A. nidulans		Acetylation	[56]
			Methylation	[57]
	S. cerevisiae	S10	Phosphorylation	[110,243,244]
	S. pombe			[245]
	S. cerevisiae	T11	Phosphorylation	[190]
	S. cerevisiae	K14	Acetylation	[39,155,191,194,230,237,243,244]
			Methylation	[155]
			Butyrylation	[246]
	C. albicans		Acetylation	[241]
	N. crassa		Acetylation	[195]
	F. graminearum		Acetylation	[45]
	A. nidulans		Acetylation	[56]

S. cerevisiae		Acetylation	[39,155,194,230]
		Methylation	[155]
		Butyrylation	[246]
C. albicans	K18	Acetylation	[195]
N. crassa		Acetylation	[195]
F. graminearum		Acetylation	[45]
A. nidulans		Acetylation	[21]
S. cerevisiae		Acetylation	[155,194]
		Methylation	[155]
		Propionylation	[219]
	K23	Butyrylation	[246]
C. albicans		Acetylation	[241]
N. crassa		Acetylation	[195]
F. graminearum		Acetylation	[193]
A. nidulans	R26	Methylation	[88]
S. cerevisiae		Acetylation	[155,194]
		Methylation	[155]
		Ubiquitylation	[201]
		Butyrylation	[219]
C. albicans	K27	Acetylation	[241]
N. crassa		Acetylation	[195]
		Methylation	[195,247]
F. graminearum		Acetylation	[45]
		Methylation	[63]
A. nidulans		Methylation	[21]
S. cerevisiae	S28	Phosphorylation	[201]
S. cerevisiae	S31	Phosphorylation	[201]
S. cerevisiae		Acetylation	[155,248]
		Methylation	[155,231,249,250]
S. pombe		Methylation	[251]
C. albicans	K36	Acetylation	[241]
N. crassa		Acetylation	[195]
		Methylation	[195,252]
A. nidulans		Methylation	[253]
S. cerevisiae		Acetylation	[188–191]
		Methylation	[254]
		Ubiquitylation	[219]
S. pombe	K37	Methylation	[241]
C. albicans		Acetylation	[195]
N. crassa		Acetylation	[195]
		Methylation	[195]
S. cerevisiae	Y41	Phosphorylation	[199,200]
S. pombe			[200]
S. cerevisiae	K42	Ubiquitylation	[255]
S. cerevisiae		Acetylation	[256–259]
		Ubiquitylation	[201]
		Malonylation	[144]
	K56	Propionylation	[219]
S. pombe		Acetylation	[260]
C. albicans		Acetylation	[241]
N. crassa		Acetylation	[195]
S. cerevisiae			[199]
S. pombe	S57	Phosphorylation	[200]
C. albicans			[198]

	S. cerevisiae<br>*S. pombe*	T58	Phosphorylation	[228]<br>[200]
	S. cerevisiae	K64	Acetylation<br>Ubiquitylation	[155]<br>[261]
	S. cerevisiae	K79	Acetylation<br>Methylation<br>Ubiquitylation<br>Succinylation	[204]<br>[262–264]<br>[201]<br>[144]
	N. crassa		Methylation	[195]
	S. cerevisiae	Y99	Phosphorylation	[265]
	S. cerevisiae	K115	Acetylation<br>Ubiquitylation	[257]<br>[266]
	S. cerevisiae	K121	Acetylation<br>Ubiquitylation	[196]<br>[255]
	S. cerevisiae	K122	Ubiquitylation	[255]
	S. cerevisiae	K125	Ubiquitylation	[255]
**H4**	*S. cerevisiae*	S1	Phosphorylation	[100,101,203]
	A. nidulans	R3	Methylation	[89]
	S. cerevisiae	K5	Acetylation<br>Sumoylation<br>Butyrylation	[191,204,267,268]<br>[135,136]<br>[219,246]
	S. pombe		Acetylation	[269]
	C. albicans		Acetylation	[270]
	N. crassa		Acetylation	[195]
	F. graminearum		Acetylation	[193]
	S. cerevisiae	K8	Acetylation<br>Sumoylation<br>Butyrylation	[204,267,271]<br>[135,136]<br>[219,246]
	S. pombe		Acetylation	[269]
	N. crassa		Acetylation	[195]
	F. graminearum		Acetylation	[193]
	S. cerevisiae	K12	Acetylation<br>Sumoylation<br>Butyrylation	[204,267,268,272]<br>[135,136]<br>[219,246]
	S. pombe		Acetylation	[269]
	C. albicans		Acetylation	[270]
	N. crassa		Acetylation	[195]
	F. graminearum		Acetylation	[193]
	A. nidulans		Acetylation	[21]
	S. cerevisiae	K16	Acetylation<br>Sumoylation	[237,267,271]<br>[135,136]
	S. pombe		Acetylation	[273]
	C. albicans		Acetylation	[270]
	N. crassa		Acetylation	[195]
	F. graminearum		Acetylation	[193]
	S. cerevisiae	K20	Methylation<br>Sumoylation	[274]<br>[135,136]
	S. pombe		Methylation	[275]
	N. crassa		Acetylation<br>Methylation	[195]<br>[195]
	F. graminearum		Methylation	[276]
	S. cerevisiae	K31	Acetylation<br>Succinylation	[204]<br>[144]
	S. pombe	S47	Phosphorylation	[277]

	C.albicans	S49	Phosphorylation	[198]
	S. cerevisiae	R55	Methylation	[219]
	S. cerevisiae	K59	Acetylation	[204]
	S. cerevisiae	S60	Phosphorylation	[222,278]
	S. cerevisiae	S64	Phosphorylation	[199,222,278]
	S. cerevisiae	K77	Acetylation<br>Succinylation<br>Malonylation	[279]<br>[144]<br>[144]
	S. cerevisiae	K79	Acetylation	[279]
	S. cerevisiae	T80	Phosphorylation	[105]
	S. cerevisiae	K91	Acetylation<br>Glutarylation	[204]<br>[280]

[1] Initiator methionine is clipped off from mature histone proteins (*e.g.*, see a) and, by convention, residue numbering does not include it. This rule for numbering has been applied here. In addition, we followed the Brno nomenclature for histone modifications, with positions referring to those used in the literature for the 'closest' model organism. Histone protein sequence alignments with numbering are provided Figure 2. [2] Not available.
